# Biocompatible Diselenide-Containing Protein Hydrogels with Effective Visible-Light-Initiated Self-Healing Properties

**DOI:** 10.3390/polym13244360

**Published:** 2021-12-13

**Authors:** Shengda Liu, Shengchao Deng, Tengfei Yan, Xin Zhang, Ruizhen Tian, Jiayun Xu, Hongcheng Sun, Shuangjiang Yu, Junqiu Liu

**Affiliations:** 1 College of Chemistry and Chemical Engineering, Central South University, Changsha 410083, China; liushengda0801@foxmail.com (S.L.); tengfeiyan@163.com (T.Y.); 2 Key Laboratory of Organosilicon Chemistry and Material Technology, Ministry of Education, College of Material, Chemistry and Chemical Engineering, Hangzhou Normal University, Hangzhou 311121, China; xujiayun@jlu.edu.cn (J.X.); sunhc@hznu.edu.cn (H.S.); 3 State Key Laboratory of Supramolecular Structure and Materials, College of Chemistry, Jilin University, Changchun 130012, China; dengsc1994@163.com (S.D.); zhangxin0422@jlenu.edu.cn (X.Z.); tianrz17@163.com (R.T.)

**Keywords:** protein hydrogel, dynamic diselenide bonds, visible light response, self-healing system

## Abstract

Smart hydrogels are typical functional soft materials, but their functional and mechanical properties are compromised upon micro- or macro-mechanical damage. In contrast, hydrogels with self-healing properties overcome this limitation. Herein, a dual dynamic bind, cross-linked, self-healing protein hydrogel is prepared, based on Schiff base bonds and diselenide bonds. The Schiff base bond is a typical dynamic covalent bond and the diselenide bond is an emerging dynamic covalent bond with a visible light response, which gives the resulting hydrogel a dual response in visible light and a desirable self-healing ability. The diselenide-containing protein hydrogels were biocompatible due to the fact that their main component was protein. In addition, the hydrogels loaded with glucose oxidase (GOx) could be transformed into sols in glucose solution due to the sensitive response of the diselenide bonds to the generated hydrogen peroxide (H_2_O_2_) by enzymatic catalysis. This work demonstrated a diselenide-containing protein hydrogel that could efficiently self-heal up to nearly 100% without compromising their mechanical properties under visible light at room temperature.

## 1. Introduction

Self-healing hydrogel materials have attracted increasing interest due to their self-healing properties for a wide range of applications in biomedical systems [1,2,3,4,5]. The choice of suitable molecular linkages for the preparation of hydrogels can meet the need for self-healing under specific conditions [6,7,8,9,10]. Dynamic covalent bonds are a special type of chemical bond that can be broken, created and reorganized in response to external stimuli, including imine, disulfide and boronic ester bonds, etc. [11,12,13,14,15]. In contrast to traditional covalent and supramolecular chemistry, dynamic covalent bonds are both dynamic and stable, a feature that has led to their widespread use in the design and preparation of new self-healing hydrogel materials, providing sufficient strength while imparting a unique dynamic character to the materials [16,17,18,19,20]. While several dynamic covalent bonds have been used to construct self-healing hydrogels, there remains a need to develop self-healing hydrogels based on novel dynamic covalent bonds with unprecedented self-healing properties for diverse applications.

Among these self-healing hydrogels based on dynamic covalent bonds, one of them employs light-induced dissociation and recombination of homolytic covalent bonds as the driving force for self-healing. The disulfide bond is one of the favorite light-driven dynamic covalent bonds, as it allows for dynamic metathesis under ultraviolet (UV) light [21,22,23,24,25]. Nevertheless, UV exposure does not provide a gentle condition, that is more energy demanding and possibly damages the system unnecessarily. Recently, Xu and co-workers have discovered that the dynamic metathesis of diselenide bonds can be easily initiated under mild conditions such as visible light, thus providing a promising opportunity for developing visible light-initiated self-healing hydrogel materials [26,27,28,29,30].

**Figure 1:**
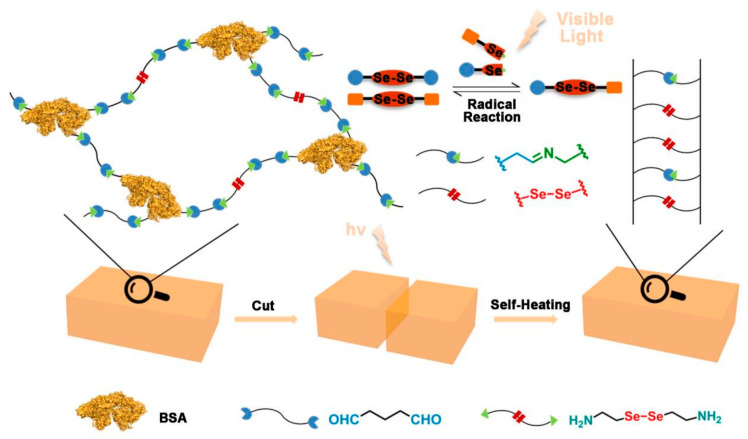
Schematic illustration of the dual dynamic bind, cross-linked protein hydrogel and the visible light-initiated self-healing of the diselenide-containing protein hydrogel.

Herein, we presented a simple method for the construction of a dual dynamic bind, cross-linked, diselenide-containing protein hydrogel, based on Schiff base bonds and diselenide bonds (Figure 1). The protein hydrogel formed through the reaction between the aldehyde groups of glutaraldehyde and the amino groups of bovine serum albumin (BSA) and selenocystamine (SeCy). The protein-based hydrogel showed excellent biocompatible and degradable performances. In addition, with a dual response in visible light, the protein hydrogel exhibited favorable self-healing properties.

## 2. Materials and Methods

### 2.1. Synthesis of the Gelator SeCy

Selenium powder (0.010 mol) and sodium borohydride (0.010 mol) were added to a three-necked flask (250 mL) and the whole system was then deoxygenated under a nitrogen atmosphere. Then, deoxygenated water (10 mL) was slowly added to the system and the reaction solution was stirred for 1 h at 40 °C until a clarified solution. After cooling down to room temperature, the obtained brownish-red solution Na_2_Se_2_ was for further use. 2-(Boc-amino)ethyl bromide (0.022 mol) in deoxygenated methanol (20 mL) was subsequently added to the above Na_2_Se_2_ solution and the reaction was stirred overnight at room temperature. The reaction stopped and the solvent was evaporated by vacuum. The product was washed 3 times with CH_2_Cl_2_ and deionized water. The organic layer was dried over anhydrous MgSO_4_. Boc-selenocystamine (Boc-SeCy) was purified by column chromatography for further use. Trifluoroacetic acid (TFA) (5 mL), with the obtained Boc-SeCy, was added to CH_2_Cl_2_ and the reaction was stirred for 4 h at room temperature. The solvent was evaporated under vacuum and ether (100 mL) was added to the system. SeCy was obtained by filtering and collecting the solids.

### 2.2. Formation of the Diselenide-Containing Protein Hydrogels

The protein BSA (210 mg) was dissolved in phosphate-buffered solution at pH 6.0 (2 mL) and mixed with 20% SeCy solution (100 μL). Then, 5% glutaraldehyde (364 μL) was homogenously mixed with the mixture. The diselenide-containing protein hydrogel was formed under visible light at the room temperature for 12 h. The heterochromatic protein hydrogels were prepared in the same way with the addition of rhodamine B and thioflavin T (0.5 mg/mL), respectively. The protein hydrogels with different selenium content were prepared in the same way by replacing part of the SeCy with hexanediamine. These chemically cross-linked hydrogels showed promising long-term stability without any apparent erosion observed.

### 2.3. Scanning Electron Microscopy Characterization

The scanning electron microscopy (SEM) image of the diselenide-containing protein hydrogel was recorded by a JEOL-JSM-6700F instrument (JEOL, Tokyo, Japan). The samples were prepared for testing in the following way. Initially, little drops of the gelator mixture were placed on a silicon wafer and the mixture gradually developed a hydrogel on the wafer. The wafer containing the hydrogel was then immediately frozen in liquid nitrogen. At last, the sample of the frozen protein hydrogel was rapidly moved to a freeze-dryer and was freeze-dried for 24 h. The sample was covered with gold spray before SEM measurement.

### 2.4. Rheological and Tensile Tests

The rheology of oscillations in diselenide-containing protein hydrogels was measured with frequency (oscillation frequency). In frequency scan tests, the frequency of the range was from 1 to 100 rad s^−1^. The prepared hydrogels were placed for testing on the platform of a rheometer MCR 302. The rheological measurements were carried out at room temperature with a 25 mm diameter parallel plate in which the stress was manipulated by the rheometer. The storage modulus (G′) test and the loss modulus (G″) test were performed under the above-described conditions.

Tensile tests were carried out using an Instron-5940 tensile instrument (Instron, Norwood, MA, USA) with a 100 N pressure measuring element. The diselenide-containing protein hydrogels were formed in strips of 3.0 mm × 4.0 mm × 12.0 mm in size. In tensile experiments, the self-repairing and tensile characteristics of the diselenide-containing protein hydrogels were measured at a rate of 20 mm min^−1^.

### 2.5. Self-Healing Tests of the Diselenide-Containing Protein Hydrogels

In the hydrogel self-healing experiments, the strips of the protein hydrogels were severed in the middle and then butt-jointed, and a little buffer solution was added dropwise to the joint interface. The spliced protein hydrogels were left in visible light or in the absence of visible light for 16 h with no additional stress or heat. The center of the illumination was 20 cm from the hydrogels during illumination. The power of the lamp was 80 W, producing a light intensity of 9.3 mW cm^−2^ at the sample position.

Following a previously reported method, the self-healing properties of the hydrogel were assessed by the ratio of the maximum stress in the self-healing hydrogel to the maximum stress in the original hydrogel [31,32].

### 2.6. Degradation Tests of the Diselenide-Containing Protein Hydrogels

The protein hydrogels produced were sensitive and responsive to redox stimuli due to the presence of diselenide bonds. Based on the responsive properties of diselenide bonds, the hydrogels were degradable by enzymatic catalysis. We prepared the diselenide-containing protein hydrogels by adding GOx at 0.5/500 mg, which produced H_2_O_2_ by oxidising glucose under mild conditions. Then, the prepared protein hydrogels containing GOx were placed in 0.2 M glucose solution and the transformation of the protein hydrogels was observed during different times.

## 3. Results and Discussion

### 3.1. Synthesis and Characterization of the Gelator

**Figure 2:**
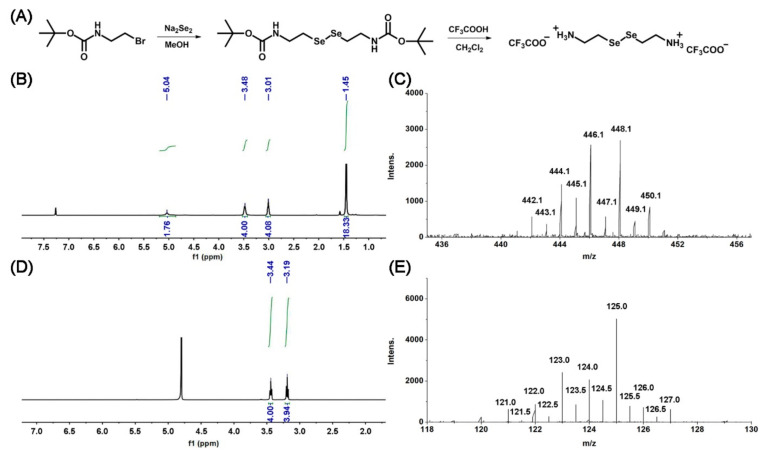
(**A**) The synthetic route of Boc-SeCy and SeCy. (**B**) ^1^H-NMR spectrum of Boc-SeCy. (**C**) ESI-MS analysis of Boc-SeCy. (**D**) ^1^H-NMR spectrum of SeCy. (**E**) ESI-MS analysis of SeCy.

The synthetic route of the gelator is illustrated in Figure 2A. SeCy was synthesized in the same way by following the previous method with a slight change [33,34]. ^1^H NMR spectrum was recorded with Bruker AVANCE III 500 (Bruker, Fällanden, Switzerland) using a tetramethylsilane (TMS) proton signal as the internal standard. ESI-MS analysis was performed by the Thermo Finnigan LCQ Advantage Mass Spectrometer (Thermo Finnigan, San Jose, CA, USA). The Boc-SeCy was firstly synthesized by the reaction of 2-(Boc-amino)ethyl bromide and Na_2_Se_2_. ^1^H NMR (500 MHz, CDCl_3_, 25 °C) δ: 5.04 (2H, CH_2_CH_2_*NH*), 3.48 (4H, SeCH_2_*CH*_2_NH), 3.01 (4H, Se*CH*_2_CH_2_NH), 1.45 (18H, OC(*CH*_3_)_3_). ESI-MS: calculated *m*/*z* 448.0, found *m*/*z* 448.1, [M]^+^ (Figure 2B,C). The SeCy was synthesized by further removal of the Boc groups from Boc-SeCy. ^1^H NMR (500 MHz, D_2_O, 25 °C) δ: 3.44 (4H, SeCH_2_*CH*_2_NH_2_), 3.19 (4H, Se*CH*_2_CH_2_NH_2_). ESI-MS: calculated *m*/*z* 125.0, found *m*/*z* 125.0, [M + 2H]^2+^ (Figure 2D,E). The results demonstrated the gelator SeCy was successfully synthesized.

### 3.2. Fabrication and Characterization of the Diselenide-Containing Protein Hydrogels

**Figure 3:**
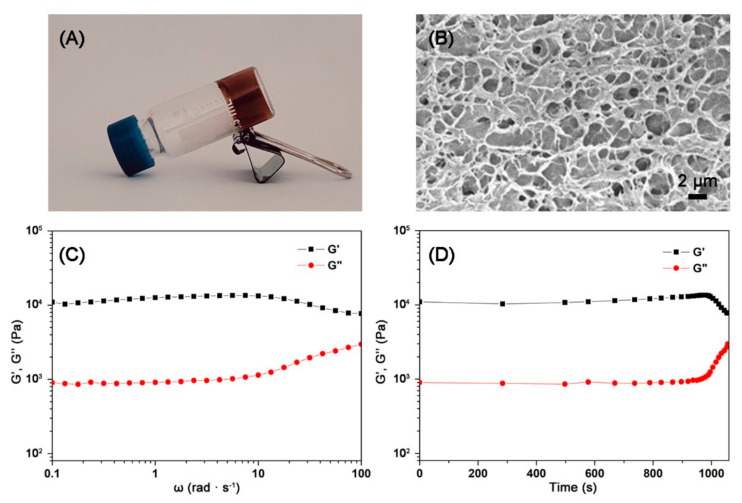
(**A**) The formation of the diselenide-containing protein hydrogel. (**B**) SEM image of the protein hydrogel. (**C**) Storage modulus (G′) and loss modulus (G″) vs. angular frequency of the protein hydrogel. (**D**) Storage modulus (G′) and loss modulus (G″) vs. time of the protein hydrogel.

The self-healing protein hydrogel with Schiff base bonds and diselenide bonds was made by the mixture of BSA with SeCy and glutaraldehyde at pH 6.0 under visible light (Figure 3A). To learn the details of the hydrogel structure in depth, SEM characterization was performed. As shown in Figure 3B, a three-dimensional (3D) network structure with numerous cavities belonging to the hydrogel was observed in SEM, which further indicated the fabrication of the hydrogel. Then, the mechanical properties of the hydrogel were determined by rheology experiments. The storage modulus (G′) is a reflection of material characteristics of energy storage and elasticity after perturbation. The loss modulus (G″) is a reflection of material properties in terms of energy loss and viscosity through relaxation or dissipation of heat. In a frequency range in which the G′ exceeded the G″ from 1 to 100 rad s^−1^, it indicated that the prepared diselenide-containing protein hydrogel was robust (Figure 3C,D).

### 3.3. Self-Healing Ability of the Diselenide-Containing Protein Hydrogels

**Figure 4:**
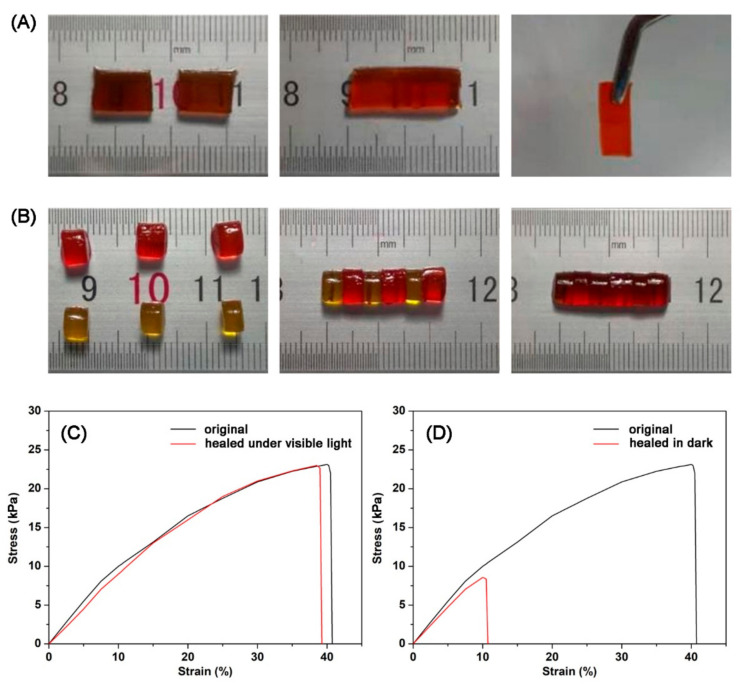
(**A**) Demonstration of multi-segment repair of hydrogels. (**B**) Demonstration of multi-segment repair of heterochromatic hydrogels. (**C**) Tensile test of original hydrogels and self-healing hydrogels under visible light. (**D**) Tensile test of original hydrogels and self-healing hydrogels in dark.

The diselenide-containing protein hydrogel was able to be self-healing by Schiff base bond exchange in an acid environment and diselenide bond exchange under visible light. In order to verify the self-healing behaviors of diselenide-containing protein hydrogels, hydrogel repair and heterochromatic hydrogel splicing experiments were performed. Firstly, a diselenide-containing protein hydrogel was divided into two halves for self-healing tests. The split hydrogel was put together and healed under visible light for 16 h, after which it was combined into a whole one (Figure 4A). Then, two diselenide-containing protein hydrogels of the same size, stained with different dyes, were prepared and cut into three separate pieces. After interconnecting the cut hydrogel pieces, they reconnected and self-healed into a complete hydrogel after visible light exposure for 16 h. In addition, the self-healing protein hydrogel had a smooth border and significant dye diffusion (Figure 4B). The results indicated that we have achieved success in constructing a self-healing hydrogel with visible light initiation, providing favorable support for our design.

To further determine the self-healing properties of the protein hydrogels quantitatively, the tensile tests were also performed. The original protein hydrogels had a high mechanical strength, reaching a strand stress of 23 kPa. The stress–strain curves in visible and shielded conditions were recorded to study the self-healing behaviors of the protein hydrogels after damage. The diselenide-containing protein hydrogels showed an outstanding self-healing effect under visible light, with a complete recovery after 16 h (Figure 4C). Nevertheless, the self-healing efficiency of the protein hydrogels was only 37% during the same time without visible light (Figure 4D). The above results indicated that the exchange of dual dynamic covalent bonds was the key to efficient self-healing of hydrogels. The synergistic effect of the dynamic exchange of Schiff base bonds and diselenide bonds under visible light achieved an efficient repair and high self-healing efficiency of the protein hydrogels with nearly 100% recovery. Furthermore, the effect of selenium content in the protein hydrogels on their self-healing capacity was investigated. In experiments, hexamethylenediamine was employed instead of partial SeCy to construct the protein hydrogels. Mechanical tests showed that with increasing hexamethylenediamine substitution and decreasing selenium content, the maximum stress of the hydrogels changed little but their self-healing rate produced a significant decrease (Figure S1). The results indicated that an increase in selenium content in a certain range of the protein hydrogels facilitated their self-healing behavior.

### 3.4. Biocompatibility Assessment and Stimulus Response of the Diselenide-Containing Protein Hydrogels

**Figure 5:**
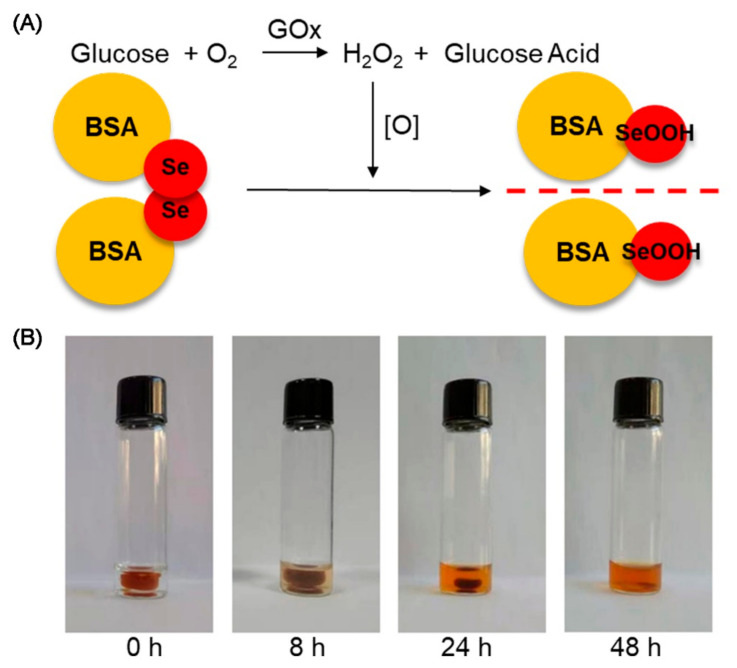
(**A**) Schematic illustration of the degradation of the GOx-loaded diselenide-containing protein hydrogels. (**B**) Degradation of the hydrogels in glucose solution in different times.

In order to study the biocompatibility of the diselenide-containing protein hydrogels, MTT assays were performed according to the previous method [35]. In vitro results showed that the survival rate of MCF-7 cells was higher than 90%, with hydrogel concentrations from 0 to 0.1 mg/ mL indicating no significant cytotoxicity of the hydrogels (Figure S2). To further investigate the role of diselenide bonds in hydrogels, we presented an approach for the degradation of diselenide-containing protein hydrogels by a stimulus response to diselenide bonds (Figure 5A). Diselenide bonds were sensitive and vulnerable to redox breakage, a property that allowed for the breakdown of chemical crosslinks in the diselenide-containing hydrogel networks and the degradation of the protein hydrogels. GOx was able to produce H_2_O_2_ by oxidizing glucose under mild conditions, and the generated H_2_O_2_ was available for diselenide bond breaking. Circular dichroism (CD) experiments firstly showed that the SeCy had no effect on the secondary structure of GOx, ensuring that the catalytic oxidation of the GOx was not affected (Figure S3). Diselenide-containing protein hydrogels, loaded with Gox, were then obtained by encapsulating GOx. After the addition of glucose solution to GOx-loaded diselenide-containing protein hydrogels, it was observed that the protein hydrogels transformed into sols over time (Figure 5B). The results showed that diselenide-containing protein hydrogels loaded with GOx were degradable by enzymatic catalysis under a mild regulation with the addition of glucose.

## 4. Conclusions

In summary, we have succeeded in constructing a dual dynamic bind cross-linked protein hydrogel, based on Schiff base bonds and diselenide bonds. The diselenide-containing protein hydrogels have highly efficient self-healing properties in visible light due to the fact that diselenide bonds allow for dynamic bond exchange via radical reactions in visible light. Under visible light, the self-healing efficiency of the hydrogel by a double dynamic response reached up to nearly 100% with no compromise in mechanical properties at room temperature, which was much higher than the self-healing efficiency by a single dynamic response of Schiff base bonds at the same time. Thus, the use of visible light in protein hydrogel systems containing diselenide bonds can optimize the healing process, shorten the healing time and improve the healing effect. The diselenide-containing hydrogel system achieved gentle visible light remote recovery of fractured hydrogel materials and it is potentially promising in the recovery of systems that are hard to access and difficult to recover in long distances. Furthermore, the diselenide-containing hydrogel loaded with GOx was degradable under mild conditions by the addition of glucose in response to the stimulus of the diselenide bonds. In addition to the desirable recoverability and degradability, the remarkable biocompatibility makes the protein hydrogels a promising application in biomimetic materials and tissue engineering.

## Data Availability

The data presented in this study are available on request from the corresponding author.

## Supplementary Materials

The following are available online at https://www.mdpi.com/article/10.3390/polym13244360/s1, Figure S1: The effect of SeCy content on the self-healing efficacy of the hydrogels during the same time, Figure S2: Cytotoxicity assessment of the diselenide-containing protein hydrogels, Figure S3: Circular dichroism analysis of GOx and GOx with SeCy.
