# Supplementary material for: Biocompatible Diselenide-Containing Protein Hydrogels with Effective Visible-Light-Initiated Self-Healing Properties
Source: Polymers (Basel). 2021 Dec 13;13(24):4360. doi: 10.3390/polym13244360 (PMC8707953; doi:10.3390/polym13244360)
Supplement: Supplementary file 1 [file polymers-13-04360-s001.zip › polymers-1483892-supplementary.pdf]

Biocompatible Diselenide-Containing Protein Hydrogels with Effective Visible-Light-Initiated Self-Healing Properties

Shengda Liu [1,2], Shengchao Deng [3], Tengfei Yan [1,2], Xin Zhang [3], Ruizhen Tian[3], Jiayun Xu [2], Hongcheng Sun [2], Shuangjiang Yu [2,*] and Junqiu Liu [2,*]

[1] College of Chemistry and Chemical Engineering, Central South University, Changsha 410083, China

[2] College of Material, Chemistry and Chemical Engineering, Key Laboratory of Organosilicon Chemistry and Material Technology, Ministry of Education, Hangzhou Normal University, Hangzhou 311121, China

[3] State Key Laboratory of Supramolecular Structure and Materials, College of Chemistry, Jilin University, Changchun 130012, China

* Correspondence: yusj@hznu.edu.cn (S.Y.); junqiuliu@jlu.edu.cn (J.L.)

Materials

Dichloromethane, ether and ethanol were purchased from Tiantai Industrial Corporation (Tianjin, China). Glutaraldehyde was purchased from Tianjin Huadong Reagent Factory (Tianjin, China). Selenium powder, sodium borohydride and trifluoroacetic acid (TFA) were Aladdin Industrial Corporation (Shanghai, China). Bovine serum albumin (BSA) was purchased from Bioengineering Ltd (Shanghai, China). Glucose oxidase (GOx) was purchased in Sigma-Aldrich (Shanghai, China). 2-(Boc-amino)ethyl bromide was purchased in Energy Chemical Ltd (Shanghai, China). Other chemical reagents were purchased from Sinopharm Group Ltd (Shanghai, China).

Instruments

^{1}H NMR spectrum was recorded with Bruker AVANCE III 500 using a tetramethylsilane (TMS) proton signal as the internal standard. ESI-MS analysis was performed by the Thermo Finnigan LCQ Advantage Mass Spectrometer. Scanning electron microscopy (SEM) images were recorded with JEOL JSM-6700F. Rheological Test was recorded with MCR 302 Rheometer. Tensile test was recorded with Instron-5940 Tensile Instrument. Circular dichroism spectrum was recorded with Bruker Biologic PMS450.

Cell culture and cell viability

MCF-7 cells were obtained from the Institute of Biochemistry and Cell Biology, Shanghai Institute for Biological Sciences, Chinese Academy of Sciences (Shanghai, China). The cell culture medium was RPMI-1640 medium containing 10% FBS and 1% antibiotics. The cell culture environment was 37 °C with 5% CO_2 in a cell culture incubator at 37 °C. MTT assay was chosen to evaluate the cytotoxicity of the diselenide-containing protein hydrogels. After 24 h of cell incubation at 37 °C, the complete medium was replaced by the medium with different concentrations of the protein hydrogels (from 0 to 0.10 mg/ mL) for another 12 h. Then the cells were added with MTT, which was reduced to an insoluble formazan crystal. After further 4 h incubation, DMSO solution was added to each well to dissolve the formazan product. The absorbance of formazan was measured at 492 nm by using a microplate reader.

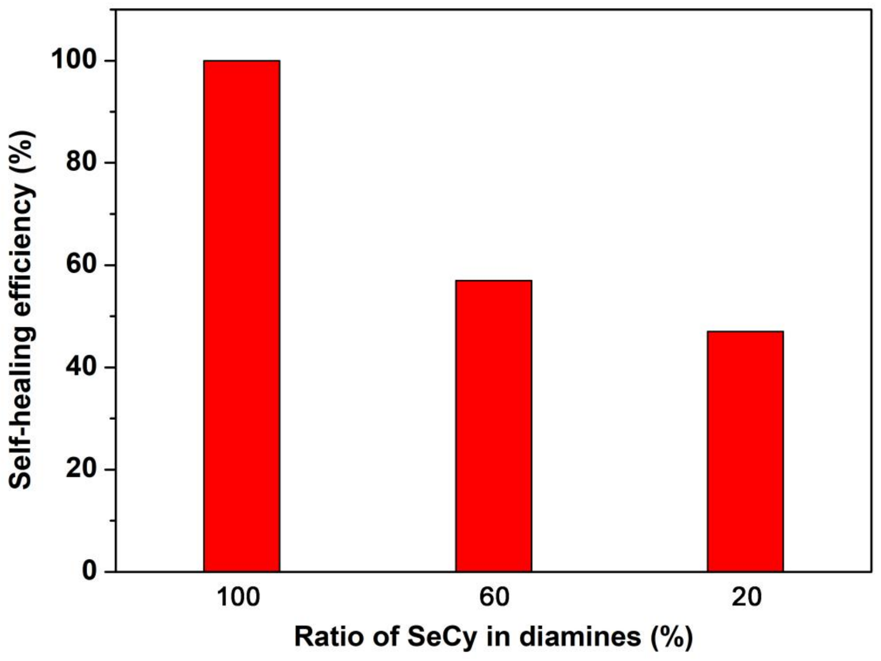

Figure S1. The effect of SeCy content on the self-healing efficacy of the hydrogels during the same time.

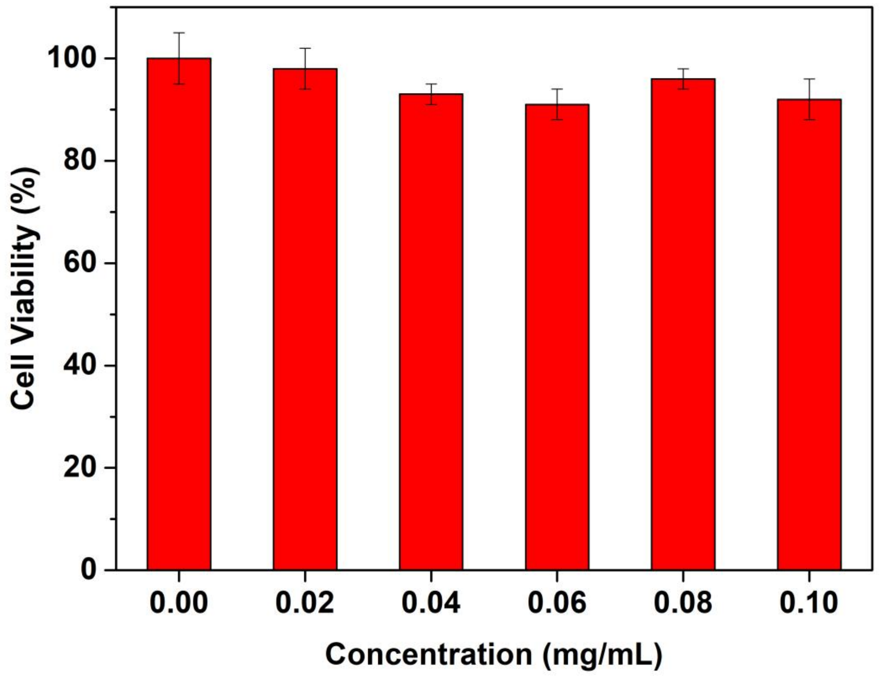

Figure S2. Cytotoxicity assessment of the diselenide-containing protein hydrogels.

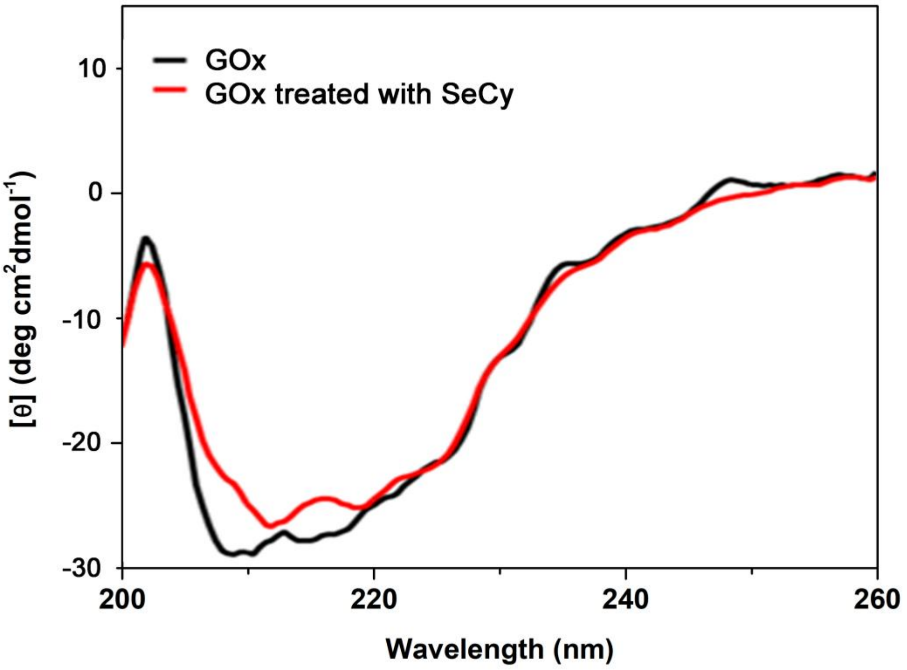

Figure S3. Circular dichroism analysis of GOx and GOx with SeCy.
